# An Intelligent Exercise Intervention for Enhancing Hot Executive Functions in Children With Attention-Deficit/Hyperactivity Disorder: A Randomized Clinical Trial

**DOI:** 10.31083/AP51439

**Published:** 2026-08-05

**Authors:** FeiLong Zhu, Xi Zhang, DongQing Kuang, ZhuFeng Li, Li Yang, YuFeng Wang, Ming Zhang, YuanChun Ren

**Affiliations:** ^1^School of Sports Medicine and Rehabilitation, Beijing Sport University, 100084 Beijing, China; ^2^College of Physical Education and Sports, Beijing Normal University, 100875 Beijing, China; ^3^Shanghai Yangzhi Rehabilitation Hospital (Shanghai Sunshine Rehabilitation Center), School of Medicine, Tongji University, 201619 Shanghai, China; ^4^College of Artificial Intelligence, Beijing Normal University, 100875 Beijing, China; ^5^NHC Key Laboratory of Mental Health (Peking University), National Clinical Research Center for Mental Disorders (Peking University Sixth Hospital), Peking University Sixth Hospital, 100191 Beijing, China; ^6^The Affiliated Xuzhou Rehabilitation Hospital of Xuzhou Medical University, Xuzhou Rehabilitation Hospital, 221009 Xuzhou, Jiangsu, China; ^7^Beijing Key Laboratory for Big Data Innovative Application of Child and Adolescent Mental Disorders, 100875 Beijing, China

**Keywords:** attention-deficit/hyperactivity disorder, intelligent exercise intervention, randomized controlled trial, hot executive functions

## Abstract

**Background:**

Deficits in hot executive functions (EFs), including risky decision-making, delay discounting, and emotion regulation, represent important yet relatively understudied features of attention-deficit/hyperactivity disorder (ADHD). Although physical exercise has been shown to improve cool EFs, its effects on hot EFs remain uncertain. Furthermore, conventional intervention approaches are often limited by accessibility and implementation barriers, underscoring the need for intelligent, home-based alternatives.

**Methods:**

In this three-arm randomized controlled trial, 145 children aged 6–10 years with ADHD were randomly assigned to an intelligent exercise group (EG1; n = 53), a traditional offline exercise group (EG2; n = 45), or a wait-list control group (CG; n = 47). Both intervention groups participated in a 12-week program consisting of three sessions per week, each lasting 45–60 minutes and integrating physical and cognitive training. Participants in EG1 completed the intervention through a custom-developed WeChat mini-program (“LeDongYing”), which incorporates computer vision–based motor assessment, dynamically personalized exercise prescriptions, real-time feedback, an artificial intelligence (AI)-assisted guidance agent, and a tailored exercise library. Participants in EG2 received coach-led, face-to-face training sessions. Outcome measures included risky decision-making, delay discounting, and emotion regulation. Intervention effects were evaluated using generalized linear mixed models, with potential confounders included as covariates.

**Results:**

For risky decision-making, EG1 demonstrated significantly greater improvements than CG in Block 3 (*β* = 3.38, *p* = 0.019), Block 4 (*β* = 6.89, *p* < 0.001), and total net score (*β* = 13.07, *p* = 0.012). EG2 also showed significantly greater improvements than CG in Block 3 (*β* = 3.49, *p* = 0.019) and Block 4 (*β* = 4.38, *p* = 0.009). No significant differences were observed between EG1 and EG2 for any risky decision-making outcome (all *p* > 0.05). For delay discounting, both EG1 (*β* = 0.12,* p* < 0.001) and EG2 (*β* = 0.12, *p* < 0.001) demonstrated significantly greater improvements than CG, with no significant difference between the two intervention groups (*β* = 0.00, *p* = 0.968).For emotion regulation, both EG1 (*β* = 3.38, *p* = 0.002) and EG2 (*β* = 2.71, *p* = 0.014) improved significantly more than CG, whereas no significant difference was observed between EG1 and EG2 (*β* = 0.66, *p* = 0.507). Adherence and participant satisfaction did not differ significantly between EG1 and EG2 (both *p* > 0.05).

**Conclusions:**

The intelligent exercise intervention appears to be an effective, scalable, and accessible non-pharmacological approach for improving hot executive functions in children with ADHD. Its efficacy was comparable to that of traditional face-to-face exercise programs, suggesting that this delivery model may help overcome implementation barriers and expand access to rehabilitation services without implying superiority over conventional approaches.

**Clinical Trial Registration:**

The study has been registered on https://www.chictr.org.cn/ (registration number: ChiCTR2200065413; registration link: https://www.chictr.org.cn/showproj.html?proj=182412).

## Main Points

1. A 12-week intelligent exercise intervention significantly improved hot executive functions in children with attention-deficit/hyperactivity disorder.

2. The intelligent exercise intervention was comparably effective to conventional face-to-face exercise training, without suggesting an advantage over conventional approaches.

3. The intelligent exercise intervention model may provide a scalable and accessible alternative for addressing barriers to conventional rehabilitation.

## 1. Introduction

Attention-deficit/hyperactivity disorder (ADHD) is one of the most common neurodevelopmental disorders in school-aged children, with an estimated global prevalence of 5.29–7.20% [1,2]. It is characterized by the core symptoms of inattention, hyperactivity, and impulsivity, and arises from the combined influence of genetic and environmental factors [3,4]. These symptoms can substantially impair academic performance, emotional functioning, and social relationships, creating a considerable burden for both children and their families [5].

In addition to its core behavioral symptoms, ADHD is strongly associated with deficits in executive functions (EFs) [6]. EFs are higher-order processes involved in the regulation of thought, behavior, and emotion [7]. Current theoretical models, including the inhibitory control model and dual-systems theory, commonly distinguish between “cool” and “hot” EFs in ADHD [7,8]. Cool EFs refer to relatively abstract and decontextualized processes, such as inhibitory control, working memory, and cognitive flexibility, and are largely linked to the dorsolateral prefrontal cortex [8,9]. These functions have been extensively studied in children with ADHD. By contrast, hot EFs involve affective decision-making, delay gratification, and emotional regulation in motivationally salient situations, and are more closely related to the orbital and medial prefrontal cortex [8,9].

Hot EF deficits are highly relevant to daily functioning in children with ADHD [8]. Difficulties in affective decision-making may increase impulsive and risky behaviors. Greater delay discounting may reduce the ability to wait for larger future rewards. Poor emotional regulation may contribute to emotional outbursts, frustration intolerance, interpersonal conflict, and difficulties in peer relationships [9]. These impairments may remain even when core ADHD symptoms are partly controlled, and they are closely related to later social, academic, and psychological outcomes [9]. However, despite their clinical importance, hot EFs have received much less attention than cool EFs in ADHD intervention research, especially in studies of non-pharmacological interventions.

Physical exercise is a promising non-pharmacological intervention for children with ADHD. Previous studies and meta-analyses have shown that structured exercise programs can improve core ADHD symptoms as well as cool EFs [10,11,12,13,14,15]. For example, our previous randomized controlled trial (RCT) and network meta-analysis found that different exercise interventions significantly improved inhibitory control, working memory, and cognitive flexibility [6,14,16]. However, evidence on the effects of exercise on hot EFs remains limited. As a result, it is still unclear whether exercise can improve affective and motivational aspects of EFs in children with ADHD.

Although traditional face-to-face exercise interventions can be effective, their implementation in real-world settings is often limited by practical barriers, such as time and space constraints, transportation demands, limited access to standardized programs, and a shortage of trained professionals [17]. Digital health technologies, including artificial intelligence (AI) and the Internet of Things (IoT), may help address these barriers. Intelligent exercise interventions delivered through smartphone-based platforms may offer a more accessible, scalable, and personalized approach while maintaining intervention fidelity and reducing logistical burden. Based on this rationale, our team developed “LeDongYing”, a WeChat mini-program designed for children with ADHD. This AI-supported intelligent exercise platform integrates computer vision-based motor skill assessment, a rule-based personalized exercise prescription algorithm, a tailored exercise library, and a large language model-powered guidance agent. These functions allow the program to deliver dynamic, home-based exercise training tailored to each child’s motor and cognitive characteristics.

This three-arm RCT aimed to examine the effects of a 12-week intelligent exercise intervention on hot EFs in children with ADHD. The primary outcomes were risky decision-making, delay discounting, and emotional regulation. The intelligent exercise group was compared with a traditional offline exercise group and a wait-list control group. We hypothesized that the intelligent exercise intervention would improve hot EFs relative to the wait-list control and would show equivalent effects to the traditional face-to-face exercise training. By focusing on the relatively understudied domain of hot EFs, this study sought to provide evidence for an accessible and scalable rehabilitation model for children with ADHD.

## 2. Materials and Methods

### 2.1 Study Design

This study was a randomized controlled trial with three arms, including two parallel intervention groups and one control group. The trial was conducted between March 2023 and December 2024. Data analysis and reporting followed the CONSORT guidelines for randomized controlled trials [18]. The study was registered as part of a broader research project. The final sample size exceeded the initially registered target because more eligible participants than anticipated were recruited during the recruitment period. Certain procedural details were also refined during study implementation, without altering the main study objectives, interventions, outcomes, or overall design.

### 2.2 Participants

From March 2023 to July 2024, 145 children diagnosed with ADHD were recruited through psychiatrist referrals and poster advertisements at Peking University Sixth Hospital. Children were eligible for inclusion if they met all of the following criteria: (1) were aged 6–10 years, regardless of sex; (2) met the diagnostic criteria for ADHD according to the Diagnostic and Statistical Manual of Mental Disorders, Fifth Edition (DSM-V) [19], including the inattentive (ADHD-I), hyperactive/impulsive (ADHD-HI), and combined (ADHD-C) subtypes; (3) had an intelligence quotient (IQ) above 80, as assessed using the Wechsler Intelligence Scale [20]; (4) were able to understand and follow instructions and were physically capable of participating in the exercise intervention; (5) if previously treated with stimulant medications, completed a washout period of at least 48 hours before testing; and (6) had no previous experience of cognitive-behavioral therapy or extracurricular sports training.

Children were excluded if they met any of the following criteria: (1) organic neurological disorders, childhood schizophrenia, pervasive developmental disorders, intellectual disability, epilepsy, autism spectrum disorder, or similar conditions; children with ADHD as the primary diagnosis were eligible even if they had common comorbidities such as oppositional defiant disorder; (2) hearing or visual impairment; (3) participation in another clinical trial that could affect the primary outcomes; or (4) comorbid conditions that would prevent safe participation in home-based exercise.

### 2.3 Sample Size Calculation

The sample size was estimated based on the assumption that the intelligent exercise intervention would produce an effect similar to that reported in a previous exercise study in children with ADHD, which found a medium effect on cool EFs (standard mean difference [SMD] = 0.61) [15]. Power analysis using G*Power (version 3.1.9.7; Heinrich-Heine-Universität Düsseldorf, Düsseldorf, Germany) indicated that 30 participants per group would be required to achieve 80% power at a significance level of *α *= 0.05. After allowing for an estimated 15% dropout rate during the 12-week intervention, the required sample size was increased to 105 participants, with 35 children in each group.

### 2.4 Randomization, Allocation Concealment, and Blinding

Participants were randomly assigned in a 1:1:1 ratio to the intelligent exercise group (experimental group 1, EG1), traditional offline exercise group (experimental group 2, EG2), or wait-list control group (control group, CG). The random sequence was generated using SPSS software (version 26.0; IBM Corp, Chicago, IL, USA) by an independent statistician who was not involved in study implementation. Group assignments were placed in sealed envelopes to ensure allocation concealment. Simple randomization without stratification was used because the planned sample size per group (≥30) was considered sufficient to maintain baseline comparability in major demographic and clinical variables. Due to the nature of the interventions, blinding of participants and intervention staff was not feasible. However, outcome assessors were blinded to group allocation and remained unaware of participants’ assignment during both baseline and postintervention assessments.

### 2.5 Interventions and Control Conditions

Participants in EG1 received individualized, intelligent exercise prescriptions through a mobile application for home-based training under parental supervision. In contrast, participants in EG2 received traditional tailored face-to-face exercise training delivered by professional coaches. Both intervention groups completed an integrated motor-cognitive exercise program designed to improve motor skills and EFs in children with ADHD. The overall exercise protocol was prescribed at low-to-moderate intensity, with the average heart rate maintained below 65% of age-predicted maximum heart rate (208–0.7×age) [21]. Both groups trained 3 times per week, with each session lasting 45–60 minutes for 12 weeks.

#### 2.5.1 Intelligent Exercise Intervention

The intelligent exercise intervention for EG1 was delivered through a WeChat mini-program, “LeDongYing”, which was independently designed and developed by our research team based on the characteristics and needs of children with ADHD. The platform adopts a closed-loop management model that includes assessment, personalized exercise prescription, intervention delivery, and reassessment. “LeDongYing” was developed over a 12-month period by a multidisciplinary team through iterative content development, technical implementation, and pilot testing. The intervention was administered at home, with 3 sessions per week and 45–60 minutes per session, under parental supervision.

The assessment module is divided into 2 age groups, 3–6 years and 7–12 years, allowing the platform to evaluate children’s motor skill development and EF status. Motor skills were assessed using a computer vision pipeline based on Google MediaPipe for human key-point extraction. Time-series body movement features were then analyzed using a random forest classifier to automatically evaluate levels of gross motor skills, fine motor skills, and balance ability.

Personalized exercise recommendations were generated using a rule-based hybrid algorithm that integrated exercise goals, age, sex, ADHD subtype, motor ability, baseline EF level, and previous training performance. The intervention design drew on multiple approaches, including motor and cognitive skill-based developmental learning and gamification. A central feature of each exercise plan was the integration of graduated physical and cognitive demands. Movement experts developed a broad range of exercises tailored to different motor ability levels in children with ADHD and aligned these activities with the children’s motor and cognitive needs. All exercises were photographed and video recorded, and each was coded according to target category and assigned 3–5 levels of difficulty. These videos, accompanied by detailed audio instructions, were uploaded to “LeDongYing”. The final exercise library included more than 300 videos targeting gross motor skills, fine motor skills, and balance ability, and it continues to be updated. Examples of training tasks included dynamic locomotor activities such as walking, running, and jumping under varying conditions to enhance both adaptability and cognitive engagement. These tasks involved path variation, including straight, zigzag, and curved movement patterns to challenge spatial planning and cognitive flexibility; pacing variation, including steady and variable speeds such as gradual acceleration, deceleration, and sudden stopping to train inhibitory control and self-regulation; and dual-task cognitive loading, such as counting steps, recalling movement sequences, or responding to auditory cues (e.g., “freeze”) to engage working memory and inhibition. A safe and unobstructed physical space was required for all intervention activities to ensure that dynamic movements, including walking, running, jumping, agility drills, and balance tasks, could be performed without hazard or restriction.

Real-time AI-based interaction was supported by a customized intelligent agent, “LeDongJingLing”, developed on the large language model. This agent provided natural language guidance, movement correction prompts, and real-time feedback during training. It also answered parents’ questions regarding exercise dosage, safety, and ADHD-related management strategies by querying a dedicated domain knowledge base built from clinical and exercise science literature.

Parents were instructed to supervise their children’s home-based training using the recommended weekly videos and prescribed exercise dosage. Adherence was automatically monitored through in-app timestamp recording, session completion logs, and synchronization with wearable devices. The system issued push notifications for incomplete sessions and tracked weekly participation rates. At the end of each training stage, “LeDongYing” displayed a pop-up message stating, “Congratulations! You have earned another LeDong Star today”. At the end of the intervention, each participant received a summary of the total number of stars earned during the study. Attendance was recorded for every training session, and children with attendance rates above 90% received a reward after completing the intervention. Through integration with wearable devices, the backend expert system was also able to monitor each child’s training process and exercise dosage and provide timely reminders. The wearable device used in this program was a self-developed smartwatch. Screenshots of the “LeDongYing” WeChat mini-program and the expert system webpage are provided in **Supplementary Material 1** and **Supplementary Material 2**, respectively.

#### 2.5.2 Traditional Exercise Intervention

Consistent with the general exercise protocol, EG2 received a 12-week integrated motor-cognitive exercise program delivered face-to-face by professional coaches at the university gymnasium of the principal investigator and at a local elementary school. All coaches completed standardized training focused on ADHD-specific behavioral guidance and intervention fidelity. Sessions were delivered according to a printed intervention manual to ensure consistency across coaches and settings. Each session followed a standardized 3-phase structure: a 5-minute warm-up consisting of light aerobic activity and joint mobilization, a 40-50-minute core training phase involving integrated motor-cognitive activities, and a 5-minute cool-down phase including static stretching and relaxation to reduce exercise-induced arousal.

Gross motor training typically began with aerobics or interactive games, followed by balance exercises, with aerobics serving as the primary mode of training. The core training phase was specifically designed to improve gross motor skills and EFs through tasks with clear cognitive demands. For example, children performed aerobic movements in coordination with music of varying rhythms, requiring them to attend to rhythm patterns, remember movement sequences, make real-time judgments in response to rhythm changes, shift between auditory and motor cues, and inhibit irrelevant distractions. Fine motor training was delivered in a gamified format and included activities such as bean picking, thread threading, and plasticine kneading, targeting unimanual coordination, bimanual coordination, and eye-hand coordination.

To ensure that exercise intensity remained within the predefined low-to-moderate range, 3 children per session were randomly selected to wear accelerometers (ActiGraph GT9X), and pulse rates were monitored regularly. Accelerometer data were sampled at 30 Hz and aggregated into 15-second epochs using ActiLife software (version 6.13.4; ActiGraph LLC, Pensacola, FL, USA). Moderate-to-vigorous physical activity (MVPA) was defined using age-specific Evenson cut-points (≥2296 counts/min), which have been validated for children aged 6–10 years. As in EG1, attendance was recorded for each session, and participants with attendance rates above 90% received a surprise gift after completion of the 12-week intervention.

#### 2.5.3 Control Conditions

Participants in the CG received minimal intervention, consisting of general guidance on physical activity and exercise provided through a downloadable electronic booklet and a single brief educational session lasting 10–15 minutes. At baseline, participants in the CG also wore accelerometers (ActiGraph GT9X) to allow objective assessment of their physical activity levels. After completion of the study, children in the CG were offered the same exercise intervention provided to the experimental groups.

### 2.6 Outcome Measures

Although “LeDongYing” included an embedded assessment module, all baseline and postintervention assessments were conducted on site by the same trained assessor to ensure consistency in testing conditions. Demographic and clinical data were collected for all participants, including age, sex, height, weight, body mass index z score (BMI z score), IQ, ADHD subtype, medication use, and physical activity level. Motor skill development was also assessed to support the development of individualized exercise prescriptions. Gross motor skills were assessed using the Test of Gross Motor Development, Third Edition (TGMD-3), which includes the 2 subdomains of locomotor skills and object control skills. In addition, the Movement Assessment Battery for Children, Second Edition (MABC-2) was used to assess manual dexterity, aiming and catching, and balance.

Hot EFs were operationalized as 3 related but distinct affective-cognitive domains: risky decision-making, delay discounting, and emotion regulation. These domains were assessed using validated, developmentally appropriate measures commonly used in research involving children with ADHD.

#### 2.6.1 Risky Decision-Making

Risky decision-making was assessed using the Hungry Donkey Task (HDT), a computerized reward-based decision-making task designed to measure temporal foresight and risky decision-making in children [22]. The task was programmed and administered using E-Prime 2.0 software (Psychology Software Tools, Inc., Pittsburgh, PA, USA) to ensure standardized stimulus presentation and precise trial timing. As a child-adapted version of the Iowa Gambling Task (IGT), the HDT requires participants to help a donkey collect apples by selecting 1 of 4 doors (A, B, C, or D). Each choice is associated with gains and losses of different magnitudes, and cumulative gains or losses are displayed visually using a colored red-green bar at the bottom of the screen. Doors A and B are disadvantageous and lead to a net loss over time, whereas doors C and D are advantageous and yield a net gain. The task includes 100 trials. Previous studies have shown that the HDT has acceptable psychometric properties, including adequate internal consistency (0.63–0.69) and construct validity [22].

Net scores were calculated by subtracting the number of disadvantageous choices (A + B) from the number of advantageous choices (C + D), with higher scores indicating better performance. To examine changes in decision-making across the task, net scores were also calculated for 5 blocks of 20 trials each.

#### 2.6.2 Delay Discounting

Delay discounting was assessed using the delay discounting task (DDT) [23], which was also programmed and administered using E-Prime 2.0 software. In this computerized task, adapted for children aged 6 to 12 years, participants were asked to make repeated choices between a smaller immediate hypothetical reward and a larger delayed reward. Choosing the smaller immediate reward advanced the task to the next trial, whereas choosing the larger delayed reward resulted in progressively longer delays for that option in subsequent trials until the participant switched to the immediate alternative. To increase task engagement, rewards were represented using images of chocolate. The smaller immediate reward was 1 piece of chocolate, whereas the larger delayed rewards were 2, 5, 10, or 20 pieces, available after delays of 1 day, 3 days, 1 week, 1 month, 3 months, 6 months, 1 year, or 3 years. The DDT has shown good test-retest reliability in children’s samples (0.69–0.76) [23].

The primary outcome for this task was the area under the curve (AUC), which was used as an index of delay discounting. AUC was calculated by summing the trapezoidal areas under the discounting curve across successive delay intervals using the following formula:


$$AUC=\sum \left(x_{2}-x_{1}\right)\times \left[\left(y_{1}+y_{2}\right)/2\right]$$

In this equation, x_1_ and x_2_ represent the proportional positions of a given delay and the next longer delay relative to the maximum delay, respectively, and y_1_ and y_2_ represent the subjective indifference points for those delays, expressed as proportions of the objective delayed reward amount. AUC values range from 0 to 1, with lower values indicating steeper discounting, greater preference for smaller immediate rewards, and greater impulsive choice.

#### 2.6.3 Emotional Regulation

Emotion regulation was assessed using the Primary School Students’ Emotional Intelligence Questionnaire, developed by Wang et al. [24], which has demonstrated good reliability and validity (0.71–0.89). The questionnaire was completed by parents based on their observations of the child’s daily behavior. It contains 40 items, of which 10 items (C01, C06, C11, C16, C21, C26, C31, C35, C37, and C39) specifically assess emotion regulation. Each item is rated on a 4-point scale: never (0), occasionally (1), often (2), and frequently (3). A raw total score for the emotion regulation subscale was obtained by summing the scores of the relevant items. This score was then converted to a percentage score ranging from 0 to 100 by dividing the raw score by the maximum possible subscale score and multiplying by 100.

#### 2.6.4 Adherence and Satisfaction

Adherence was compared between EG1 and EG2 over the 12-week intervention period using the total number of completed training sessions and the proportion of participants who achieved full attendance (36 sessions, corresponding to 3 sessions per week for 12 weeks). Parental satisfaction was assessed using a 5-point Likert scale, with response options scored as follows: very satisfied (5), somewhat satisfied (4), neutral (3), somewhat dissatisfied (2), and very dissatisfied (1).

### 2.7 Statistical Analyses

Demographic and baseline characteristics were summarized as means ± standard deviations (SDs) for continuous variables and as numbers (percentages) for categorical variables. In accordance with the CONSORT statement, no statistical tests were performed to compare baseline characteristics between groups [25]. Missing data were handled using multiple imputation. Missing values in outcomes and covariates were assumed to be missing at random and were imputed using predictive mean matching, with 10 imputed data sets generated. All analyses followed the intention-to-treat (ITT) principle, whereby all randomized participants were analyzed in their originally assigned groups regardless of intervention adherence or session completion. Changes in outcome measures from baseline to postintervention were analyzed using generalized linear mixed models (GLMM). Each model included treatment group (EG1, EG2, and CG), time (baseline and postintervention), and the group × time interaction as fixed effects, with participant ID included as a random intercept to account for within-subject correlation. Age, sex, IQ, ADHD subtype, MVPA, and medication use were included as covariates, selected a priori based on their established associations with executive functioning in children with ADHD. Standardized *β* coefficients are reported for GLMM results. Post hoc pairwise comparisons between groups were conducted using estimated marginal means, and the Benjamini-Hochberg false discovery rate (FDR) method was applied to adjust for multiple comparisons. The total number of completed training sessions and parental satisfaction scores were compared between EG1 and EG2 using independent-samples *t* tests. The proportion of participants who completed all intervention sessions was compared using the chi-square test. Comparisons between EG1 and EG2 were conducted using 2-sided tests without a prespecified noninferiority margin. Therefore, the absence of statistically significant differences between the 2 intervention groups was interpreted as comparable efficacy rather than formal noninferiority. A 2-sided *p* value of less than 0.05 was considered statistically significant. All statistical analyses were performed using R software (version 4.4.0; R Foundation for Statistical Computing, Vienna, Austria).

## 3. Results

### 3.1 Participant Characteristics

**Fig. 1.**
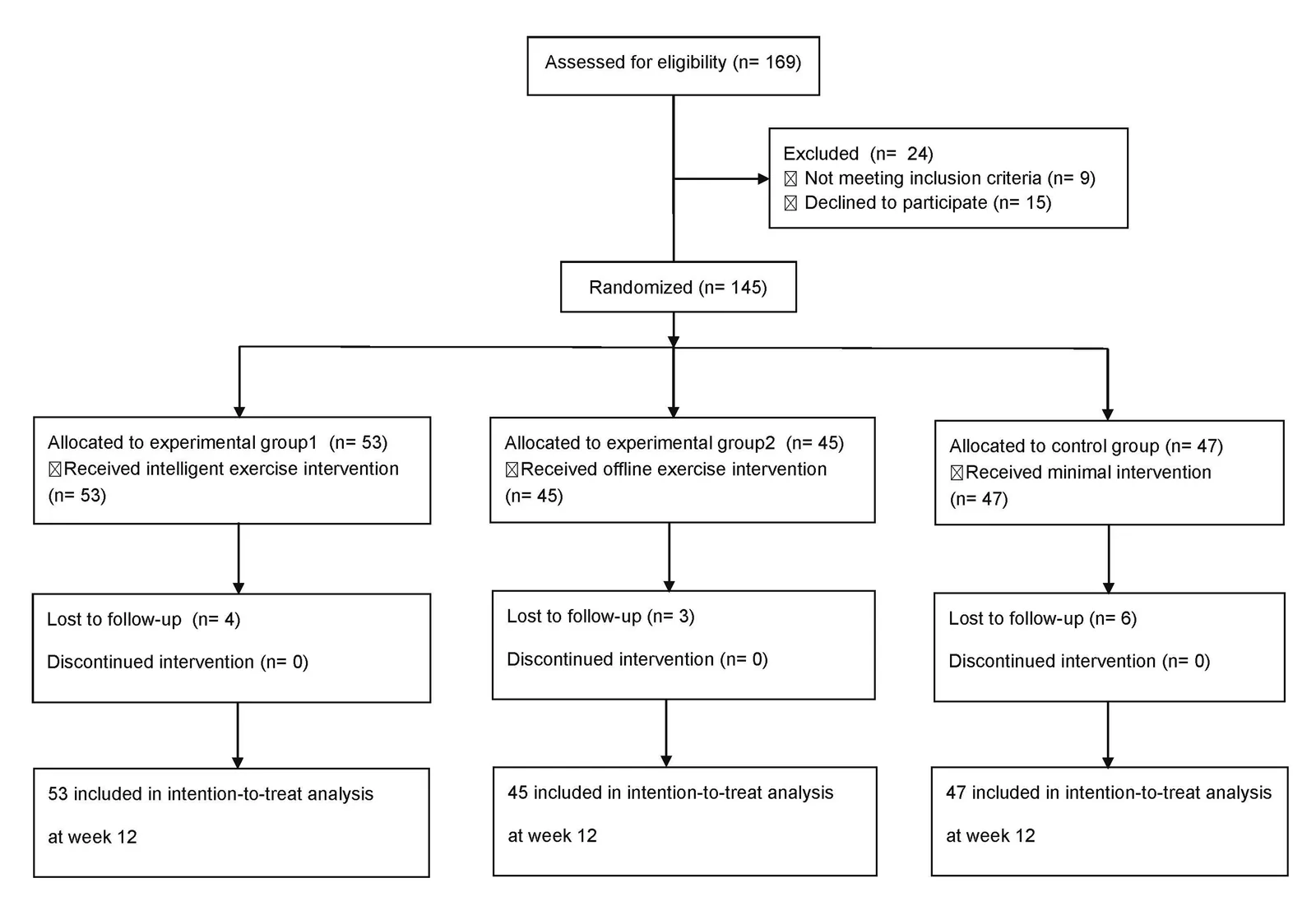
**Patient flow diagram of trial participation**.

A total of 169 children with ADHD were initially screened for eligibility. Of these, 24 were excluded: 9 did not meet the inclusion criteria and 15 declined to participate. The remaining 145 eligible participants were randomly assigned to EG1 (n = 53), EG2 (n = 45), or the CG (n = 47). The participant flow throughout the study is presented in Fig. 1. Baseline demographic and clinical characteristics, including age, sex, height, weight, BMI z score, ADHD subtype, core symptom severity, medication history, daily MVPA, and baseline performance in risky decision-making, delay discounting, and emotion regulation, are summarized in Table 1. During the 12-week intervention period, 13 participants were lost to follow-up, including 4 in EG1, 3 in EG2, and 6 in the CG. No significant differences in demographic characteristics were observed among participants who dropped out across the 3 groups (all *p *> 0.05). All 145 randomized participants were included in the ITT analysis.

**Table 1.** **Descriptive characteristics of the participants**.

Characteristic		EG1 (n = 53)	EG2 (n = 45)	CG (n = 47)
Age (years)		8.45 (1.08)	8.76 (1.27)	8.43 (1.28)
	6–8 years	34 (64.15%)	24 (53.33%)	31 (65.96%)
	9–10 years	19 (35.85%)	21 (46.67%)	16 (34.04%)
Sex				
	Female	8 (15.09%)	5 (11.11%)	6 (12.77%)
	Male	45 (84.91%)	40 (88.89%)	41 (87.23%)
Height (cm)		133.52 (9.12)	136.88 (10.44)	134.09 (8.92)
Weight (kg)		30.66 (8.06)	32.21 (8.86)	30.49 (7.63)
BMI z score		–0.06 (2.09)	–0.16 (1.92)	–0.20 (2.03)
IQ		102.89 (9.90)	101.84 (12.12)	105.40 (11.34)
Subtype				
	ADHD-I	34 (64.15%)	25 (55.56%)	25 (53.19%)
	ADHD-HI	1 (1.89%)	2 (4.44%)	1 (2.13%)
	ADHD-C	18 (33.96%)	18 (40.00%)	21 (44.68%)
Core symptom score				
	Total	31.45 (7.40)	30.40 (7.29)	31.46 (7.81)
	Inattention	17.58 (4.28)	17.91 (3.87)	18.07 (4.20)
	Hyperactivity/impulsivity	13.87 (5.26)	12.49 (5.89)	13.39 (5.67)
Medicine intake				
	None	48 (90.57%)	41 (91.11%)	39 (82.98%)
	Yes	5 (9.43%)	4 (8.89%)	8 (17.02%)
MVPA (minutes/day)		76.33 (44.06)	75.71 (44.14)	71.69 (36.59)
TGMD-3 scores				
	Locomotion	29.04 (6.11)	29.78 (7.99)	28.60 (5.84)
	Object control	33.13 (8.29)	34.09 (8.47)	33.51 (8.52)
MABC-2 scores				
	Manual dexterity	6.83 (3.09)	6.76 (2.55)	7.43 (3.14)
	Aiming and catching	8.06 (3.99)	7.18 (2.29)	7.79 (3.72)
	Balance	7.47 (3.04)	6.42 (2.08)	6.81 (2.95)
Risky decision-making				
	Block 1 net score	–2.19 (6.73)	–0.09 (5.85)	–0.55 (7.53)
	Block 2 net score	–10.19 (5.81)	–8.04 (6.64)	–10.68 (6.00)
	Block 3 net score	–9.51 (6.42)	–8.13 (5.66)	–7.11 (5.92)
	Block 4 net score	–6.94 (9.25)	–3.91 (8.85)	–6.77 (8.95)
	Block 5 net score	0.57 (11.40)	2.71 (9.90)	2.60 (13.01)
	Total net score	–28.26 (28.59)	–17.47 (23.42)	–22.51 (32.95)
Delay discounting		0.48 (0.16)	0.48 (0.16)	0.45 (0.16)
Emotional regulation		46.58 (16.13)	47.89 (16.99)	50.62 (16.47)

Data were presented as the means (SD), n (%). EG1, experimental group 1; EG2, experimental group 2; CG, control group; BMI, body mass index; IQ, intelligence quotient; ADHD-I, attention-deficit/hyperactivity disorder inattentive subtype; ADHD-HI, attention-deficit/hyperactivity disorder hyperactive-impulsive subtype; ADHD-C, attention-deficit/hyperactivity disorder combined subtype; MVPA, moderate-to-vigorous physical activity; TGMD-3, Test of Gross Motor Development-3rd edition; MABC-2, Movement Assessment Battery for Children-2nd edition; SD, standard deviation.

### 3.2 Effects on Risky Decision-Making

In Blocks 1 and 2, no significant pre-post changes were observed in any group, and no significant between-group differences were found (*p *> 0.05). Significant improvements emerged from Block 3 onward in both EG1 and EG2.

In Block 3, the mean net score increased significantly from –9.51 (SD 6.42) to –4.64 (SD 6.55) (MD = 4.87 [2.88 to 6.86], *p *< 0.001) in the EG1, and the mean net score increased significantly from –8.13 (SD 5.66) to –3.16 (SD 7.39) (MD = 4.98 [2.61 to 7.35], *p *< 0.001) in the EG2. Significant changes were also found in the CG (MD = 1.49 [0.19 to 2.79], *p *= 0.026). Compared with the CG, both EG1 (*β *= 3.38, SE 1.32, *p *= 0.019) and EG2 (*β *= 3.49, SE 1.38, *p *= 0.019) showed significantly greater increases from baseline to endpoint. No significant difference was found between EG1 and EG2 (*β *= –0.11, SE 1.34, *p *= 0.934).

In Block 4, the mean net score increased significantly from –6.94 (SD 9.25) to –0.60 (SD 7.74) (MD = 6.34 [3.88 to 8.80], *p *< 0.001) in the EG1, and the mean net score increased significantly from –3.91 (SD 8.85) to –0.09 (SD 6.21) (MD = 3.82 [1.88 to 5.76], *p *< 0.001) in the EG2. No significant change was found in the CG (MD = –0.55 [–2.60 to 1.50], *p *= 0.590). Compared with the CG, both EG1 (*β *= 6.89, SE 1.51, *p *< 0.001) and EG2 (*β *= 4.38, SE 1.57, *p *= 0.009) showed significantly greater improvement. The difference between EG1 and EG2 was not significant (*β *= 2.52, SE 1.53, *p *= 0.101).

In Block 5, the mean net score increased significantly from 0.57 (SD 11.40) to 4.30 (SD 7.40) (MD = 3.74 [1.43 to 6.04], *p *= 0.002) in the EG1, and the mean net score increased significantly from 2.71 (SD 9.90) to 5.87 (SD 6.72) (MD = 3.16 [1.03 to 5.29], *p *= 0.005) in the EG2. No significant change was found in the CG (MD = 1.32 [–1.15 to 3.79], *p *= 0.288). However, no significant between-group differences were found for change in Block 5 net score (*p *> 0.05).

For the total net score, the mean value increased significantly from –28.56 (SD 28.59) to –12.30 (SD 13.26) (MD = 15.96 [9.74 to 22.18], *p *< 0.001) in the EG1, and from –17.47 (SD 23.42) to –5.91 (SD 15.04) (MD = 11.56 [4.78 to 18.33], *p *= 0.001) in the EG2. No significant change was found in the CG (MD = 2.89 [–3.75 to 9.53], *p *= 0.385). Compared with the CG, EG1 showed a significantly greater increase in total net score from baseline to endpoint (*β *= 13.07, SE 4.48, *p *= 0.012), whereas the difference between EG2 and the CG did not reach statistical significance (*β *= 8.66, SE 4.66, *p *= 0.098). No significant difference was observed between EG1 and EG2 (*β *= 4.41, SE 4.53, *p *= 0.332).

**Fig. 2.**
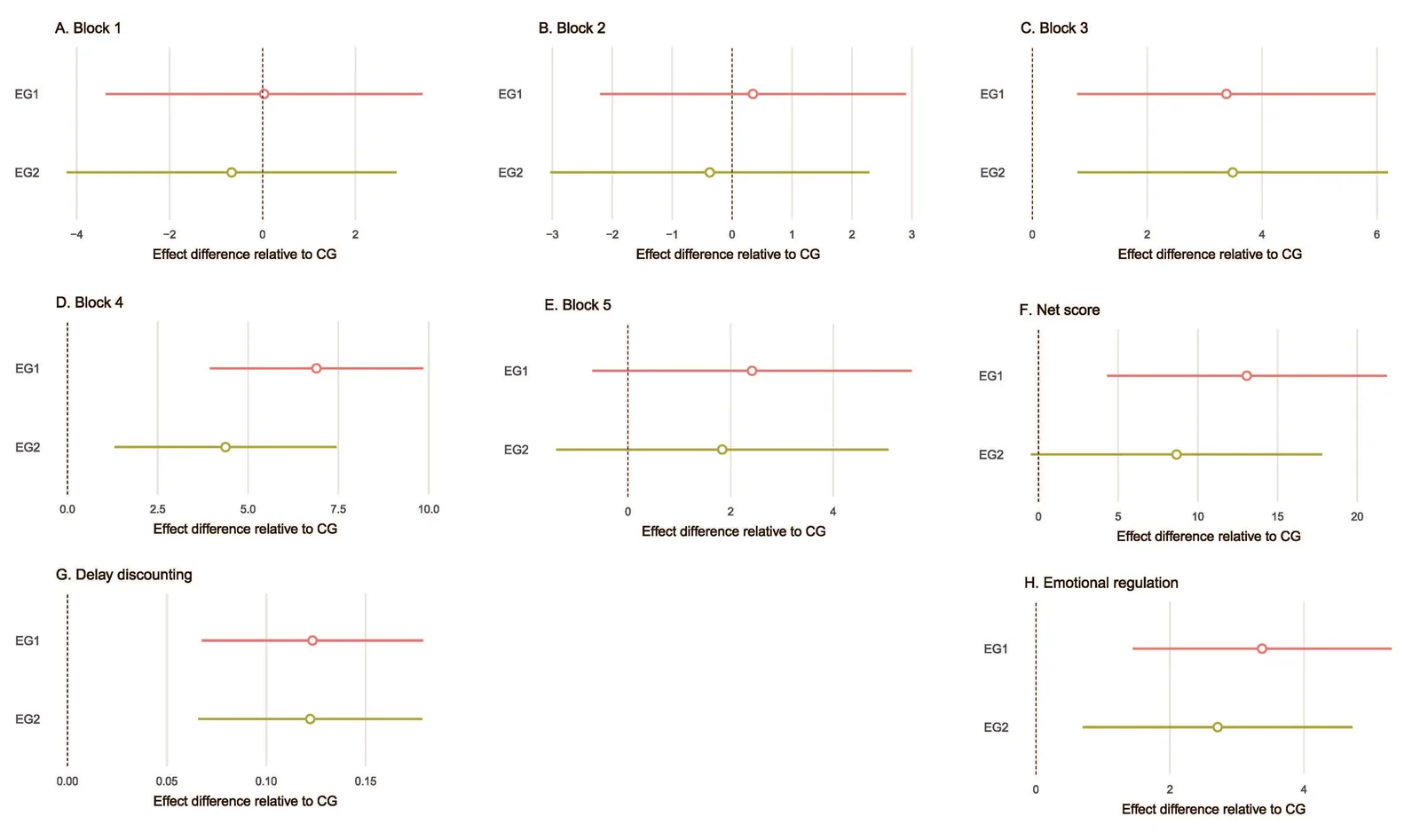
**Intervention effects on decision-making and emotional regulation outcomes**. The effect difference in Risky decision-making (A–F), delay discounting (G), and emotional regulation (H) relative to the CG.

All within-group comparisons are presented in Table 2, and between-group comparisons are shown in Fig. 2A–F.

**Table 2.** **Baseline and postintervention hot executive functions data for three groups**.

Outcome measure		Baseline	Endpoint	Mean difference (95% CI)	*p*
Risky decision-making					
Block 1 net score					
	EG1	–2.19 (6.73)	–2.11 (6.76)	0.08 (–1.89 to 2.04)	0.939
	EG2	–0.09 (5.85)	–0.71 (6.09)	–0.62 (–3.59 to 2.35)	0.674
	CG	–0.55 (7.53)	–0.51 (5.41)	0.04 (–2.69 to 2.78)	0.975
Block 2 net score					
	EG1	–10.19 (5.81)	–9.25 (4.21)	0.94 (–1.03 to 2.92)	0.341
	EG2	–8.04 (6.64)	–7.82 (4.73)	0.22 (–1.91 to 2.36)	0.835
	CG	–10.68 (6.00)	–10.09 (4.13)	0.60 (–0.94 to 2.13)	0.439
Block 3 net score					
	EG1	–9.51 (6.42)	–4.64 (6.55)	4.87 (2.88 to 6.86)	<0.001
	EG2	–8.13 (5.66)	–3.16 (7.39)	4.98 (2.61 to 7.35)	<0.001
	CG	–7.11 (5.92)	–5.62 (5.02)	1.49 (0.19 to 2.79)	0.026
Block 4 net score					
	EG1	–6.94 (9.25)	–0.60 (7.74)	6.34 (3.88 to 8.80)	<0.001
	EG2	–3.91 (8.85)	–0.09 (6.21)	3.82 (1.88 to 5.76)	<0.001
	CG	–6.77 (8.95)	–7.32 (6.02)	–0.55 (–2.60 to 1.50)	0.590
Block 5 net score					
	EG1	0.57 (11.40)	4.30 (7.40)	3.74 (1.43 to 6.04)	0.002
	EG2	2.71 (9.90)	5.87 (6.72)	3.16 (1.03 to 5.29)	0.005
	CG	2.60 (13.01)	3.91 (7.29)	1.32 (–1.15 to 3.79)	0.288
Total net score					
	EG1	–28.56 (28.59)	–12.30 (13.26)	15.96 (9.74 to 22.18)	<0.001
	EG2	–17.47 (23.42)	–5.91 (15.04)	11.56 (4.78 to 18.33)	0.001
	CG	–22.51 (32.95)	–19.62 (16.04)	2.89 (–3.75 to 9.53)	0.385
Delay discounting					
	EG1	0.48 (0.16)	0.61 (0.15)	0.14 (0.09 to 0.18)	<0.001
	EG2	0.48 (0.16)	0.61 (0.14)	0.14 (0.10 to 0.18)	<0.001
	CG	0.45 (0.16)	0.47 (0.13)	0.01 (–0.01 to 0.04)	0.318
Emotional regulation					
	EG1	46.58 (16.13)	48.98 (15.00)	2.40 (1.19 to 3.60)	<0.001
	EG2	47.89 (16.99)	49.62 (14.91)	1.73 (0.28 to 3.18)	0.020
	CG	50.62 (16.47)	49.64 (14.51)	–0.98 (–2.65 to 0.70)	0.245

Data were presented as the means (SD).

### 3.3 Effects on Delay Discounting

For delay discounting, the mean value of AUC increased significantly from 0.48 (SD 0.16) to 0.61 (SD 0.15) (MD = 0.14 [0.09 to 0.18], *p *< 0.001) in the EG1, and the mean value of AUC increased significantly from 0.48 (SD 0.16) to 0.61 (SD 0.14) (MD = 0.14 [0.10 to 0.18], *p *< 0.001) in the EG2. No significant change was found in the CG (MD = 0.01 [–0.01 to 0.04], *p *= 0.318). Compared with the CG, both EG1 (*β *= 0.12, SE 0.03, *p *< 0.001) and EG2 (*β *= 0.12, SE 0.03, *p *< 0.001) showed significantly greater increases in AUC from baseline to endpoint. No significant difference was found between EG1 and EG2 (*β *= 0.00, SE 0.03,* p *= 0.968). These results are presented in Table 2 and Fig. 2G.

### 3.4 Effects on Emotional Regulation

For emotional regulation, the mean score increased significantly from 46.58 (SD 16.13) to 48.98 (SD 15.00) (MD = 2.40 [1.19 to 3.60], *p *< 0.001) in the EG1, and the mean score increased significantly from 47.89 (SD 16.99) to 49.62 (SD 14.91) (MD = 1.73 [0.28 to 3.18], *p *= 0.020) in the EG2. No significant change was found in the CG (MD = –0.98 [–2.65 to 0.70], *p *= 0.245). Compared with the CG, both EG1 (*β *= 3.38, SE 0.99, *p *= 0.002) and EG2 (*β *= 2.71, SE 1.03, *p *= 0.014) showed significantly greater increases in emotional regulation scores from baseline to endpoint. No significant difference was observed between EG1 and EG2 (*β *= 0.66, SE 1.00, *p *= 0.507). These results are shown in Table 2 and Fig. 2H.

### 3.5 Adherence and Satisfaction

During the 12-week intervention period, the mean number of completed training sessions was 29.90 (SD 4.67) in EG1 and 30.69 (SD 4.05) in EG2, with no significant between-group difference (*t *= 0.857, *p *= 0.394). Regarding full adherence to the intervention protocol, 12 participants (24.49%) in EG1 and 5 participants (11.90%) in EG2 completed all 36 scheduled sessions, and the difference was not statistically significant (χ^2^ = 2.358, *p *= 0.125).

Parental satisfaction scores were also similar between groups. The mean satisfaction score was 3.57 (SD 0.96) in EG1 and 3.76 (SD 0.98) in EG2, with no significant between-group difference (*t *= 0.946, *p *= 0.347).

### 3.6 Adverse Events

No serious adverse events were reported during the study. One minor adverse event occurred in EG2, where a child experienced a mild left ankle sprain during a training session. The participant resumed training after 3 days of rest.

## 4. Discussion

In this three-arm RCT, both the 12-week intelligent exercise intervention delivered through the “LeDongYing” app and the traditional face-to-face exercise intervention produced comparable improvements in hot EFs in children with ADHD. Relative to the wait-list control group, both active interventions significantly improved risky decision-making, delay discounting, and emotion regulation. No significant differences were observed between EG1 and EG2 across any of the outcome measures. These findings suggest that structured, cognitively enriched physical activity can improve hot EFs in children with ADHD, and that a home-based intelligent delivery model may be as effective as a conventional in-person approach.

Both intervention groups showed significant improvements in risky decision-making, particularly from Block 3 onward on the HDT. This pattern suggests improved feedback-based learning and more adaptive choice adjustment over time. In other words, children in both exercise groups appeared to become better able to use prior outcomes to shift from disadvantageous to advantageous choices. Early performance on the HDT is thought to reflect a transition from ambiguous to more explicit risky decision-making, a process that depends on the integration of affective and cognitive information [26]. The observed improvement may therefore reflect enhanced cognitive flexibility and reinforcement learning processes promoted by the integrated motor-cognitive exercise format [27,28]. It is also notable that the difference in total net score between EG2 and the control group did not reach statistical significance (*p *= 0.098). This may be because the total score aggregates performance across all task blocks, which may dilute the more pronounced between-group differences observed in the later learning phases. Greater score variability and possible mild floor effects may also have contributed.

Both intervention groups also showed significant increases in AUC on the delay discounting task, indicating a reduced tendency to devalue delayed rewards. This finding suggests improvement in impulse control and future-oriented decision-making [29]. This is particularly relevant in ADHD, as steep delay discounting is widely considered a behavioral manifestation of impulsivity [30]. Previous studies have suggested that exercise may improve inhibitory control by enhancing prefrontal cortical function and strengthening connectivity between prefrontal and limbic systems, which in turn may reduce impulsive preference for immediate rewards [30,31]. In addition, the intervention itself required children to follow routines and tolerate delayed reinforcement, such as earning stars only after session completion. This repeated practice may have supported transfer to performance on the delay discounting task.

Emotion regulation also improved significantly in both intervention groups compared with the control group. This suggests that the exercise programs had beneficial effects on children’s ability to regulate emotions in everyday contexts. Several pathways may explain this finding. First, physical exercise may reduce emotional reactivity by modulating physiological arousal and stress. Second, the cognitive demands embedded in the exercises, such as following movement sequences and inhibiting dominant responses, may have engaged neural systems that overlap with those involved in emotional control, including the medial prefrontal cortex and amygdala [32]. Third, the supportive structure of the intervention, together with parental involvement in the intelligent exercise group, may have created additional opportunities for emotional learning and regulation in daily life.

The observed gains in hot EFs are likely to reflect interacting neurobiological and psychological mechanisms. At the neurobiological level, exercise has been shown to influence dopaminergic and noradrenergic systems, which are central to reward processing, reinforcement learning, and affective decision-making [6]. These changes, particularly within frontostriatal circuitry, may improve sensitivity to reward contingencies and support adaptive strategy updating [33], consistent with the progressive improvement observed on the HDT from Block 3 onward. Exercise may also enhance prefrontal control over limbic responses, thereby strengthening the ability to delay immediate gratification in favor of larger future rewards [32]. At the psychological level, the structured exercise routines required repeated practice in self-regulation, persistence, and delayed reinforcement. The intervention also integrated physical and cognitive demands, such as rhythm coordination, sequence memory, and response inhibition, which may have facilitated transfer to affective control in daily life [34]. Together, these mechanisms provide a plausible framework linking structured exercise to improvements in hot EFs in children with ADHD.

Several additional issues should be considered when interpreting the findings and considering wider implementation. First, the intelligent exercise group used a novel digital platform, and a novelty effect may have increased initial engagement or motivation independently of the intervention content. However, the sustained gains over the 12-week period, together with the comparable outcomes observed in EG1 and EG2, suggest that the observed effects were unlikely to be explained by novelty alone. Second, parental involvement differed between the 2 intervention groups. In EG1, parents supervised home-based training, whereas in EG2, sessions were delivered directly by coaches. This difference may have influenced outcomes in EG1 through greater supervision, parent-child interaction, or support in the home environment. Future studies should measure parental involvement more directly and examine whether it moderates intervention effects. In addition, the broader implementation of AI-supported exercise interventions raises ethical and practical considerations. Data privacy and security are essential. In this study, the “LeDongYing” mini-program operated within the encrypted WeChat ecosystem, and user data were anonymized and stored on secure servers in accordance with national data protection requirements. Informed consent also explicitly covered data collection and use. At the same time, this intervention required access to a smartphone and a basic level of digital literacy among caregivers. These requirements may limit generalizability and introduce selection bias, particularly for families with fewer technological resources. Although intelligent delivery may reduce geographic and transportation barriers, it could also widen inequities if access to devices, internet connectivity, or digital skills is unevenly distributed. Future implementation studies should therefore consider strategies such as device support, simplified interfaces, and formal cost-effectiveness analyses to support equitable and sustainable adoption.

### Limitations

Several limitations should also be acknowledged. First, this was a single-center study, and participants were recruited from a specific geographic area, which may limit generalizability. Second, although outcome assessors were blinded, participants and parents were not, which may have introduced performance or reporting bias. Third, the study did not include long-term follow-up, and the durability of the observed effects therefore remains uncertain. Fourth, although baseline MVPA was included as a covariate, we did not directly assess cardiorespiratory or muscular fitness. Fifth, the proposed mechanisms were inferred from existing literature rather than directly tested in the present study. Future work should incorporate neuroimaging, electrophysiological, or other biomarker-based measures to clarify the neural correlates of behavioral change. Finally, although adherence was similar between groups, home-based training in EG1 could not be controlled as strictly as coach-led sessions in EG2.

Despite these limitations, the study has several important clinical and practical implications. Although parental supervision played a larger role in EG1 and therapist guidance was central in EG2, both delivery models produced comparable benefits. This suggests that the structured exercise content itself may have been the main active ingredient, rather than the specific delivery format. The intelligent exercise intervention therefore represents a potentially effective, scalable, and accessible nonpharmacological option for children with ADHD, particularly for those who face barriers to attending conventional face-to-face services. It may be used either as a primary intervention option or as a maintenance strategy following initial in-person training. More broadly, the integration of AI and computer vision in “LeDongYing” provides a practical model for personalized home-based rehabilitation. Future studies should validate these findings in multicenter settings, examine longer-term outcomes, explore potential differences across ADHD subtypes, and refine algorithm-based personalization using real-time data from wearable sensors.

## 5. Conclusions

This randomized controlled trial showed that 12 weeks of intelligent exercise intervention and traditional face-to-face exercise training produced comparable improvements in hot executive functions in children with ADHD. Both interventions were significantly more effective than the wait-list control condition, and no significant differences were found between the 2 active intervention groups. These findings support the intelligent home-based model as a clinically effective, scalable, and accessible nonpharmacological option for addressing barriers associated with conventional rehabilitation.

## Data Availability

The datasets used and analyzed during the current study are available from the corresponding authors on reasonable request.
