# Supplementary material for: An Intelligent Exercise Intervention for Enhancing Hot Executive Functions in Children With Attention-Deficit/Hyperactivity Disorder: A Randomized Clinical Trial
Source: Alpha Psychiatry. 2026 Aug 5;27(4):51439. doi: 10.31083/AP51439 (PMC13540014; doi:10.31083/AP51439)

Supplementary file1

A screenshot taken from the LeDongYing app

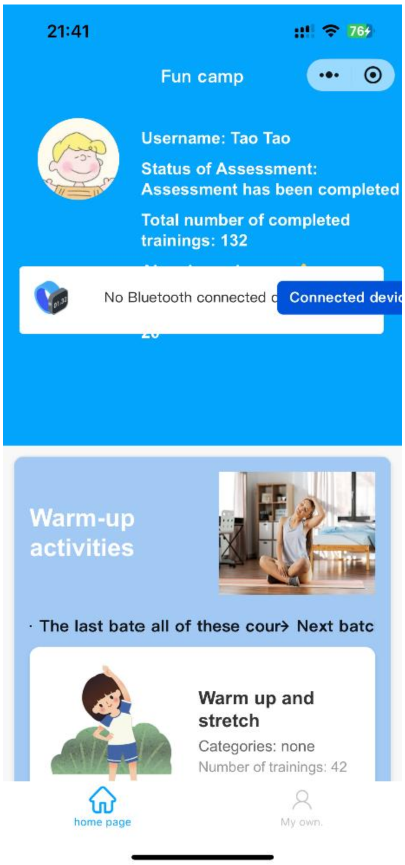

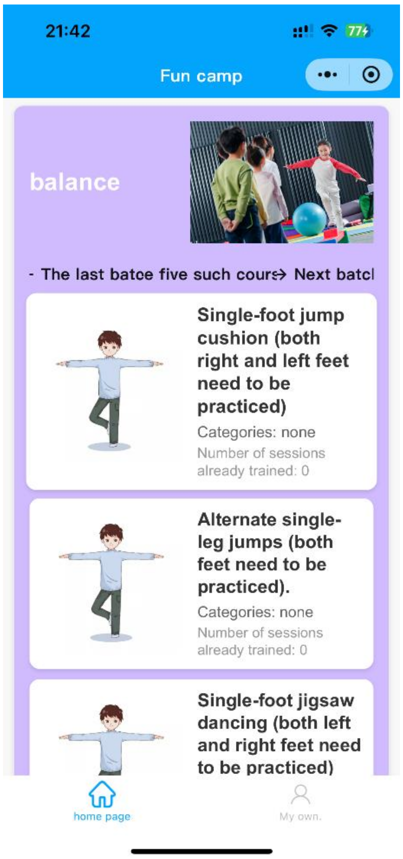

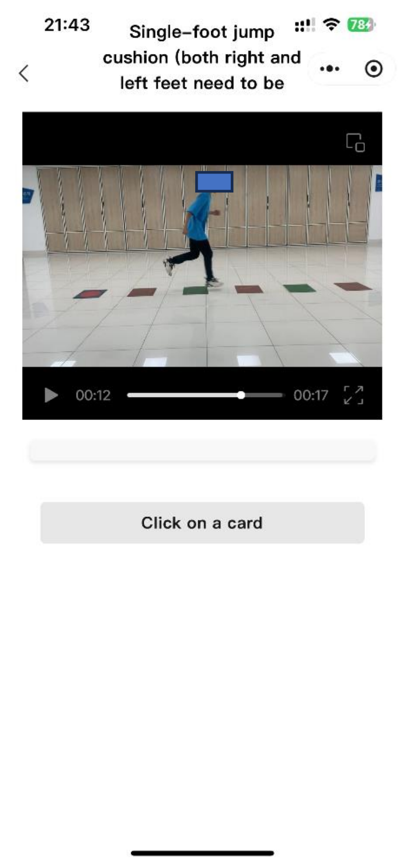

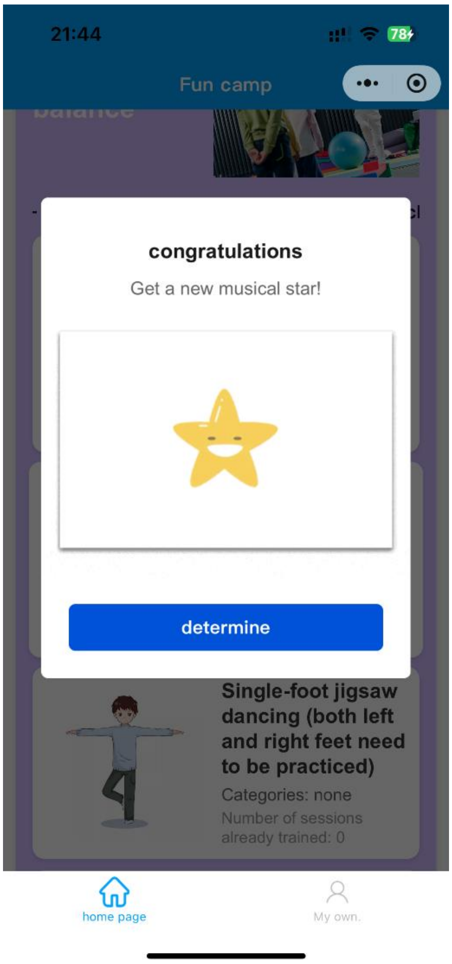

Supplement: Supplementary file 1 [file 2757-8038-27-4-51439-s1.zip › Supplementary Material 1.pdf]
